# Applying the transfer learning models on the dataset on the effect of diseases on Nagvel-betel (Piper betle) leaves

**DOI:** 10.1016/j.dib.2025.111987

**Published:** 2025-08-19

**Authors:** Milind Gayakwad, Rahul Joshi, Tulshihar Patil, Pratvina Talele, Gurunath S. Waghale, Rajendra Pawar, Nidhi Poonia, Sachin Kadam, Priyanka Paygude

**Affiliations:** a Bharati Vidyapeeth (Deemed to be University) College of Engineering, Pune; b Symbiosis Institute of Technology Pune, Symbiosis International (Deemed University), Pune, India; c School of Computer Science and Engineering, Dr. Vishwanath Karad, MIT World Peace University, Pune, India; d Sri Balaji University, Pune, India; e MIT Art, Design and Technology University, Pune, India; f Assistant Professor, Bharati Vidyapeeth Institute of Management and Information Technology, Navi Mumbai, India; g Professor, Institute of Management and Entrepreneurship Development, Bharati Vidyapeeth (Deemed to be University), Pune, India

**Keywords:** Classification, Indian Betel-Nagvel Leaf dataset, Transfer learning, Computer vision

## Abstract

The dataset of Betel leaves includes 4156 leaves affected by various diseases. These diseases include Leaf Spot, Powdery Mildew, Anthracnose, Bacterial Blight, Cercospora Leaf Spot, Sooty Mold, Downy Mildew, Wilt Disease, Rust Disease, Mosaic Virus, Black Rot, Root Rot, Stem Canker, Leaf Curl Disease, and Fusarium Wilt. The camera is used to collect high-resolution images to ensure the exact detection of the images to detect diseases. The resolution of the photos was 3000 × 4000, consuming approximately 3 mb. The data set covers a wide range of diseases, and many samples were collected under each category. The dataset is saved using a hierarchical data structure, as the name of the folder indicates the label or category of the image. The reuse and recreation of this type of dataset are ensured by mapping the name of the disease with the apparent characters of the disease on the leaves. The experiment was performed using Vision Transformer Models to check the robustness of the dataset. The result of the classification report states that the range of accuracy varies from 0.7 to 0.9.

Specifications Table

**Table:** 

Subject	Agriculture Science, Computer Vision
Specific subject area	Plant Pathology, Deep Learning
Type of data	Images
Data collection	The dataset of Betel Leaves includes 4156 images of leaves with a resolution of 3000 × 4000 that are unhealthy. The images are collected from various farms, covering a variety of diseases on Betel Leaves. It shows the signs of a disease symptom in leaves. These categories include Deformed, Cut, Brown Spot, Light-colored, Fungus, White Patches (Powder), and combinations of these categories. Note that the dataset contains 15 used categories. The Dataset is used to identify the symptoms of a disease infection in leaves. The classes are Deformed Leaf, Leaf with Cut, Leaf with Brown Spot, Leaf Light coloured, Fungus, Leaf with White Patches (Powder), and their combinations. The number of categories. These available features are mapped with the associated diseases - These diseases include Leaf Spot, Powdery Mildew, Anthracnose, Bacterial Blight, Cercospora Leaf Spot, Sooty Mold, downy mildew, Wilt Disease, Rust Disease, Mosaic Virus, Black Rot, Root Rot, Stem Canker, Leaf Curl Disease, and Fusarium Wilt.
Data source location	*Shetfale, Taluka – Aatpadi, District – Sangli* *Pin – 415306* *Maharashtra, India* *Longitude and Latitude: 17.316980, 74.933571*
Data accessibility	Repository name: Dataset on the effect of diseases on Nagvel-betel (Piper betle) leaves Data identification number: doi: 10.17632/6zzbm9dsg7.1 Direct URL to data: https://data.mendeley.com/datasets/6zzbm9dsg7/1 Instructions for accessing these data: Download the folder
Related research article	*None*

## 1 Value of the Data

• **Betel-Nagvel- There is no dataset** available on the category of the Betel. This helps to understand the exact diseases of these types of Betel Leaves.
• The dataset covers the **front and back sides of the leaves**, which is necessary as the vine reaches certain heights; the back side of the leaves is easily visible comparatively. So, the analysis based on both sides provides the option to analyze the disease effectively.
• The dataset provides a wide range of **high-quality 4156 images** for analyzing the images. Data Exploration and analysis are done by annotating the **primary apparent features** and the **Secondary**- **combination of these features**.
• **Easier reproduction** of the dataset is ensured by making the feature-wise labeling instead of the disease-wise annotation. The person with less expertise (**Modular Approach**) can prepare the dataset as per the availability of **size, shape, and color**, and then the expert from the agricultural field can map these features to the respective disease. The easier interpretation of this type of dataset is ensured by mapping the name of the disease with the apparent characters of the disease on the leaves.
• The dataset performs well for all four Models of Vision Transformers and conforms to the **robustness** of the dataset.

## 2 Background

**Fig. 1:**
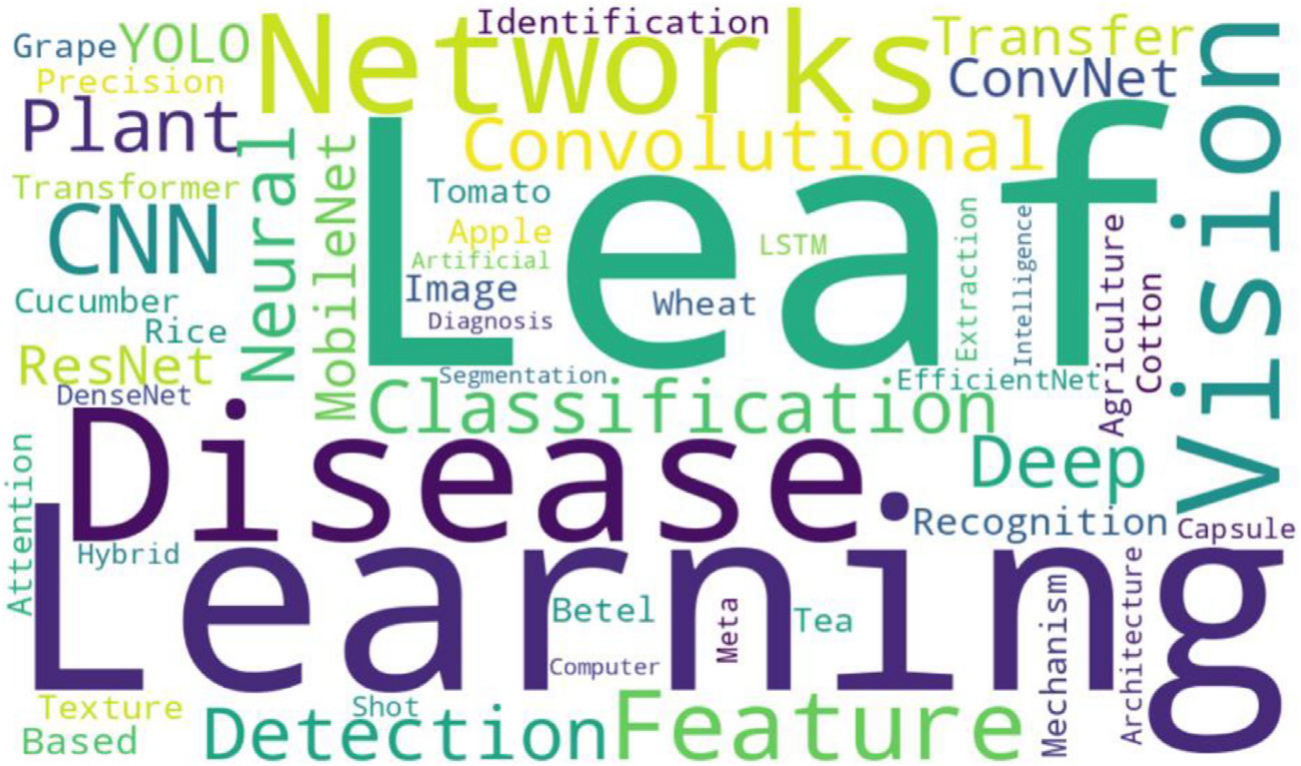
Word cloud of the keywords in reference papers used in the Article (Directly or Derived Form).

The country has exported 1,620.06 metric tons of Betel Leaves to the world, worth Rs. 42.91 crores (approximately $ 5.18 million) in 2023-24. India has exported 10,636.87 MT of Betel Nuts and Areca Nuts to the world, worth Rs. 400.13 Crore/ 48.35 USD million in 2023-24. Piper beetle (Nagvel-betel) is a widely cultivated plant with significant economic and medicinal value [1,2]. However, various diseases affect its growth and productivity, leading to substantial losses [[3], [4], [5]]. Early and accurate detection of these diseases is crucial for maintaining plant health [6]. This study presents a dataset that provides high-resolution images of diseased Piper beetle leaves to facilitate the development of machine learning-based plant disease detection models [7,8]. Fig. 1 indicates the word cloud of the keywords in reference papers.

Several studies have been conducted on plant disease detection using image processing and deep learning techniques [9,10]. Traditional methods, such as manual inspection, are labour-intensive and prone to errors [11,12]. Recent advancements in deep learning, including Convolutional Neural Networks (CNNs) and Vision Transformer Models, have significantly improved disease classification accuracy [13,14]. However, there is a lack of publicly available, high-quality datasets specific to *Piper beetle* diseases. This dataset aims to fill that gap by providing a categorized collection of disease-affected leaves [15].

The early and accurate detection of plant diseases is crucial for ensuring agricultural productivity and food security [16,17]. Deep learning techniques, particularly Convolutional Neural Networks (CNNs), have revolutionized plant disease identification by enabling the automated, precise, and efficient analysis of leaf images [18,19]. This survey focuses on applying prominent deep learning architectures—ResNet, ConvNeXtB, MobileNet, and InceptionV3—and using ImageNet for disease identification in betel leaves [20].

ResNet (Residual Network) is renowned for its deep architecture and effective feature extraction capabilities. Studies have demonstrated its high accuracy in detecting various plant diseases [21]. For instance, research has shown that ResNet achieves superior performance in plant disease classification tasks, highlighting its potential applicability to betel leaf disease identification. ResNet has proven effective in feature extraction for plant disease classification. Researchers compared their improved MobileNetV3 model with ResNet18 and found that MobileNetV3 outperformed ResNet18 in terms of accuracy for tea leaf disease identification [22,23]. Although specific studies on betel leaves using ResNet are limited, its deep feature extraction capabilities make it a promising model for this application [24]. ConvNeXtB is a modernized architecture that combines the strengths of traditional CNNs and transformer models. While its application in plant disease detection is emerging, it has shown promise in various computer vision tasks. Further research is needed to explore its effectiveness, specifically for betel leaf disease identification.

MobileNet is designed for mobile and embedded vision applications, offering a balance between accuracy and computational efficiency [25]. Studies have successfully employed MobileNet for plant disease detection, achieving high accuracy with reduced computational requirements. This makes it a suitable candidate for real-time betel leaf disease monitoring in resource-constrained environments. MobileNet has been widely used for plant disease detection due to its lightweight architecture and high computational efficiency. The authors developed a hybrid MobileNetV2-Incep-M model for classifying rice plant diseases, achieving high accuracy with minimal computational resources [26]. Similarly, the Author proposed an improved MobileNetV3 model that integrates a Coordinate Attention mechanism for tea leaf disease detection, improving accuracy to 95.88%. These studies indicate that MobileNet is a strong candidate for betel leaf disease identification. InceptionV3 is known for its deep architecture and efficient computation, making it suitable for complex image recognition tasks [27]. It has been effectively utilized in plant disease detection, demonstrating high accuracy across various datasets. Its application to betel leaf disease identification could leverage these strengths for improved diagnostic accuracy.

While direct studies on betel leaf disease identification using these architectures are limited, the success of deep learning models in related plant disease detection tasks suggests their potential applicability [28]. For example, research has demonstrated the effectiveness of CNN-based models in identifying diseases in various plant species, indicating that similar approaches could be adapted for betel leaves [29].

Despite the promising results, challenges remain in applying deep learning models to betel leaf disease identification. These include the need for large, annotated datasets specific to betel leaves, addressing variability in disease symptoms, and ensuring model generalization across different environmental conditions. Future research should focus on collecting comprehensive betel leaf datasets, exploring the integration of advanced architectures like ConvNeXtB, and developing models that balance accuracy with computational efficiency for practical deployment [30].

The application of deep learning models, such as ResNet, ConvNeXtB, MobileNet, and InceptionV3, along with transfer learning from ImageNet, holds significant promise for disease identification in betel leaves. While direct research in this area is limited, the success of these models in related plant disease detection tasks provides a strong foundation for future studies. Addressing existing challenges through dedicated research efforts will be crucial in developing effective and practical solutions for betel leaf disease management.

## 3 Data Description

Piper beetle (Nagvel-betel) is a widely cultivated plant with significant economic and medicinal value. However, various diseases affect their growth and productivity, leading to substantial losses. Early and accurate detection of these diseases is crucial for maintaining plant health and preventing large-scale crop damage. The ability to automate disease detection using deep learning models can significantly aid farmers and agricultural researchers in monitoring plant health effectively. This study presents a dataset that provides high-resolution images of diseased Piper beetle leaves to facilitate the development of machine learning-based plant disease detection models.

**Fig. 2:**
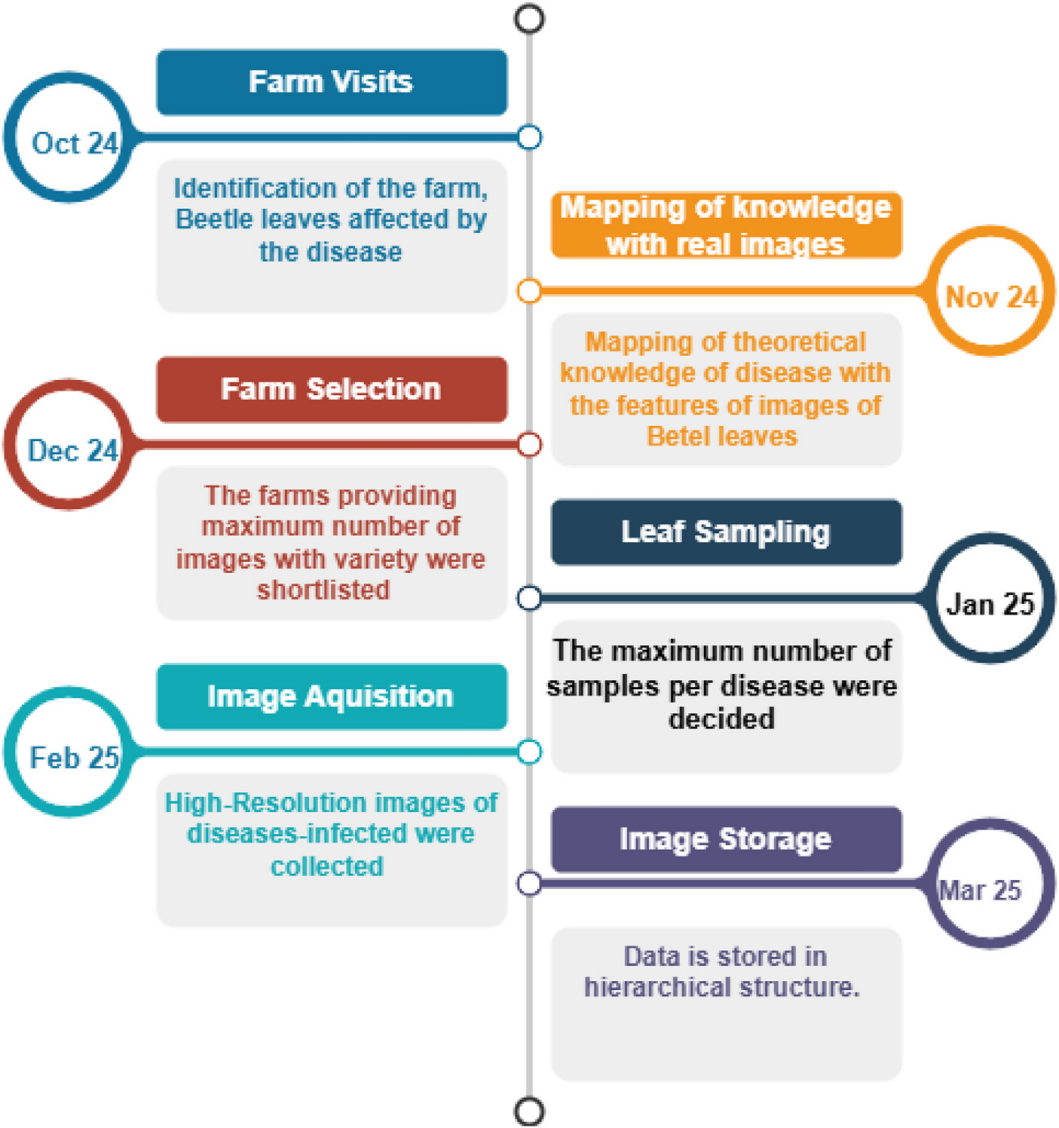
Data Acquisition Timeline.

Fig. 2 indicates the timeline from Farm selection to image storage. In October 2024, the farms where the Betel Nagvel was available were visited. In November 2024, the mapping of the identity of the diseases available and features of the disease-prone images was completed. In December 2024, The Farm was selected, and pictures of the leaves covering a maximum number of diseases were shortlisted. Sampling of the images of the leaves from all the categories of the diseases was ensured in January 2025. In February 2025, images were collected from the respected farm. The photos were stored in a hierarchical structure for effective access.

### 3.1 The resolution of the images

All images of the disease-affected leaves are collected using a SAMSUNG Galaxy S23 Mobile, Model Name – SM-S911B/DS. The dataset contains high-resolution images with the dimensions of 3000 × 4000, with the approximate size of 3-4mb for each image. The image has to be resized for further processing, so the resizing of the image to 500 × 375 consumes 11kb.

The dataset includes 15 categories of diseases with 28 folders describing the features. The categories can also be abstracted into 15 categories by considering the various objects as if they were the same (images of the Leaf from the front and back side).

The authors performed the annotations. The annotation is approved by the Consultant, BSc (Agri), who is the consultant for the Betel crop in the region. Once the images were collected, the manual validation was performed to remove the images containing noise. The pre-processing was carried out to streamline the annotation, followed by the Exploratory Data Analysis. The distribution, noise, class imbalance, Correlation of features, Feature Selection, etc., were completed.

The uploaded image has a resolution of 375 × 500 pixels, totalling 187,500 pixels, which is significantly lower than the Actual Images. The resizing affects the quality of the image, but there is no loss of the features, colours, sizes, shapes, etc.

### 3.2 Evaluation of resolution and quality

1. Image Resolution
  i) 375 × 500 px is already close to standard resized input sizes used in deep learning (For Example - 224 × 224).
  ii) However, it’s not ideal if you plan to extract fine-grained visual features such as texture, powdery patterns, or leaf margin details.
2. Visual Clarity
  i) The leaf details are soft/blurry, especially around the lesion area. The details are enough to recognize the shape, colour, or size.
  ii) Color gradients (yellow to green) are visible, but disease-specific textures/spots are not sharp. But it can be easily recognized.
3. Lossy Nature on Resize

If resized further (which is not the case in the Betel dataset) (e.g., to 128 × 128), this would severely reduce feature clarity. Since the image is already compressed in terms of both size and quality, resizing becomes lossy, affecting both resolution and visual content.

### 3.3 The data storage

**Fig. 3:**
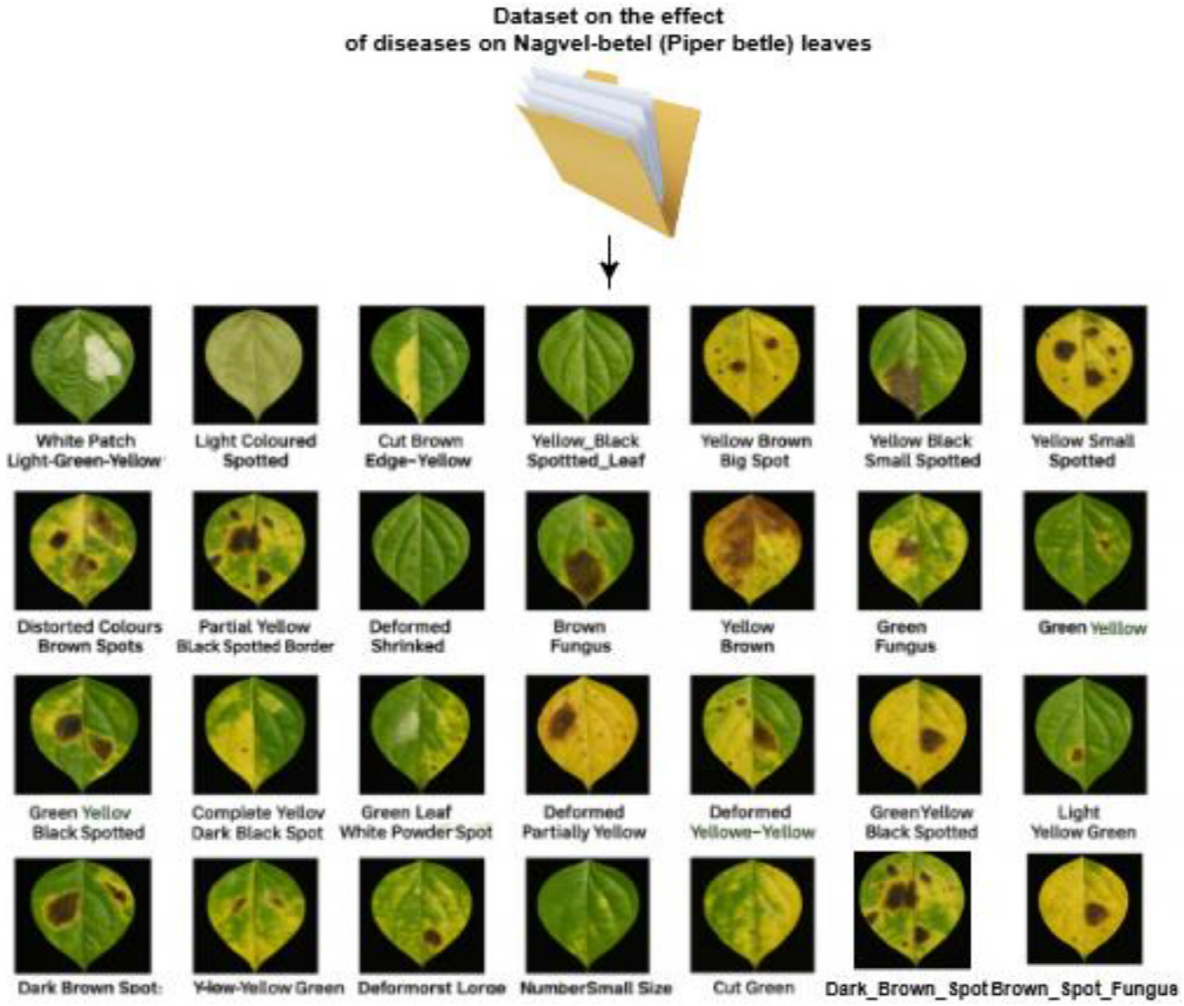
Directory Structure of the Betel Leaves Dataset.

**Fig. 4:**
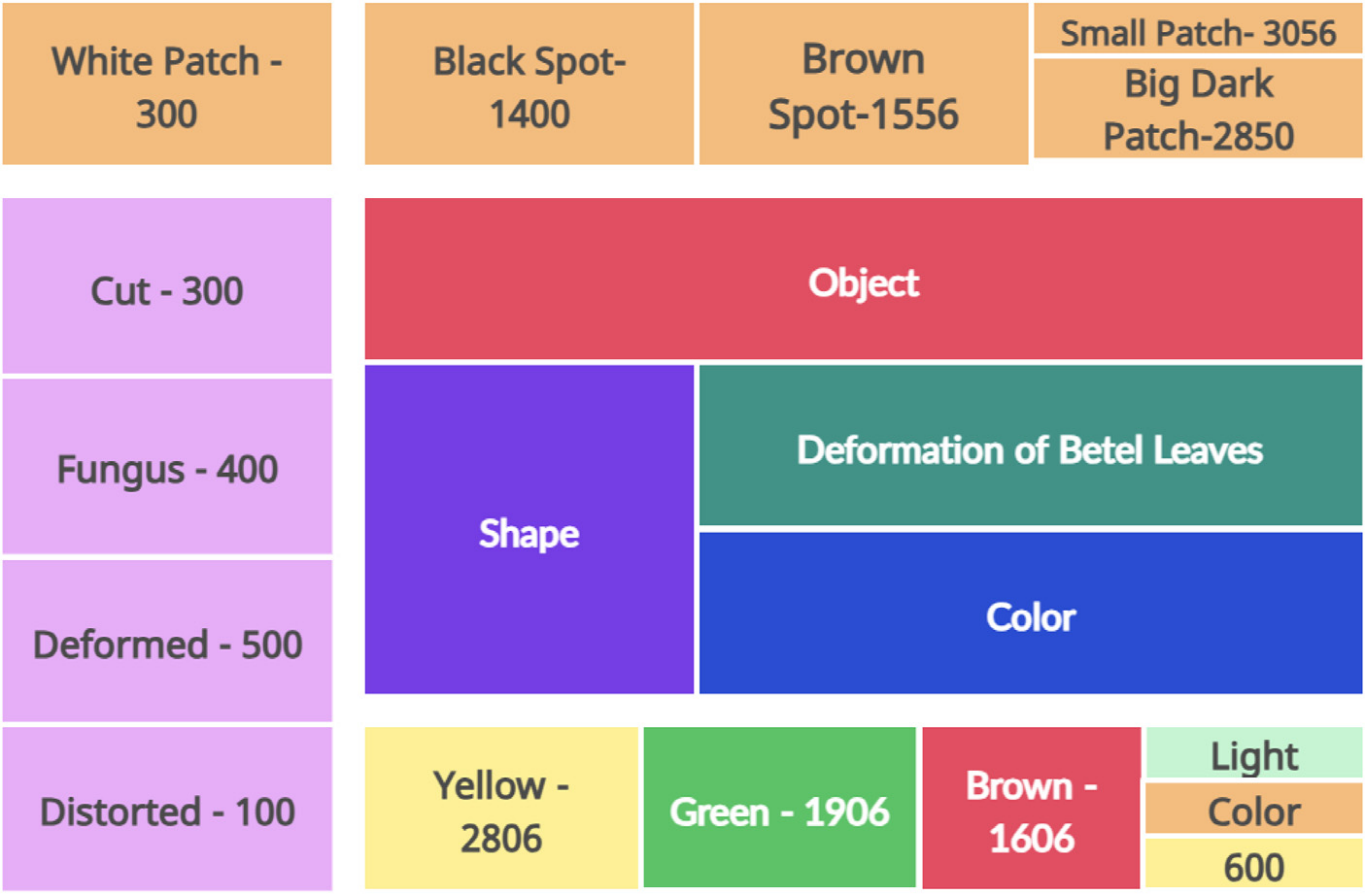
Data Exploration - Basic Features (Exclusive but hypothetical) and distribution analysis.

The directory structure helps in quickly accessing and analyzing the dataset. The mapping between the categorization from the simple primary property to secondary intersecting properties is mentioned in Fig. 3, Fig. 4, and Table 1 represents the data exploration in the form of a directory structure. While the feature and the number of samples collected from each category. The representation of the directory structure with

**Table 1:** Actual Features (Combination of Basic Features).

Dataset Analysis of the specific categories			Count
Features	Count	Features	
White_Patch_Light_Green_Yellow_Spotted	200	Green_Leaf_White_Powder_Spot	100
Light_Coloured_Spotted	200	Deformed_Yellow_Leaf_Blackspots_Edge	200
Cut_Brown_Edge_Yellow	100	Deformed_Partially_Yellow	100
Yellow_Black_Spottted_Leaf	200	GreenYellow_Black_Spotted	200
Yellow_Brown_Big_Spot	200	Dark_Brown_Spot_Edge_Yellow_Green	156
Yellow_black_Small_Spotted	100	Yellow_Green_Brown_Spotted_Cut	200
Distorted _Colours_Brown_Spots	100	Yellow_Black_Spotted_Fungus_Back	100
Partial_Yellow_Black_Spotted_Border	200	Brown_Spot_Fungus_Light_Colored	100
Deformed_Shrinked_Green	200	Light_Yelow_Green_Less_Brown_Spot	100
Brown_Fungus	200	Light_Yellow_Green_Dark_Spot	100
Yellow_Brown_Deformed	200	Light_Yellow_Green	100
Green_Fungus	200	Brown_Spot_Large_Number_Small_Size	100
Green_Yellow_Black_Spotted	201	Cut_Green_Yellow	100
Complete_Yellow_Dark_Black_Spot	200	Dark_Brown_Spot_Light_Colored	50

## 4 Experimental Design, Materials, and Methods

### 4.1 Experimental design

Data exploration helps you to better understand the data. Analyzing the data leads to an accurate technique for experimenting. The Experiment includes arranging the data in the dataset, mapping the features to the respective disease, designing the Vision Transformers, and implementing the classification report to validate the dataset.

### 4.2 Data exploration

The data acquisition process utilized image collection from vineyards and subsequent systematic preparation before adding them to the dataset.

Fig. 4 indicates the basic features necessary to decide the identity of the diseases, but there is a combination of multiple basic features that can be practically associated with the diseases. The primary features include the deformations in the leaves because of features like Size, Shape, and Color.

### 4.3 Distribution of samples across the classes

**Fig. 5:**
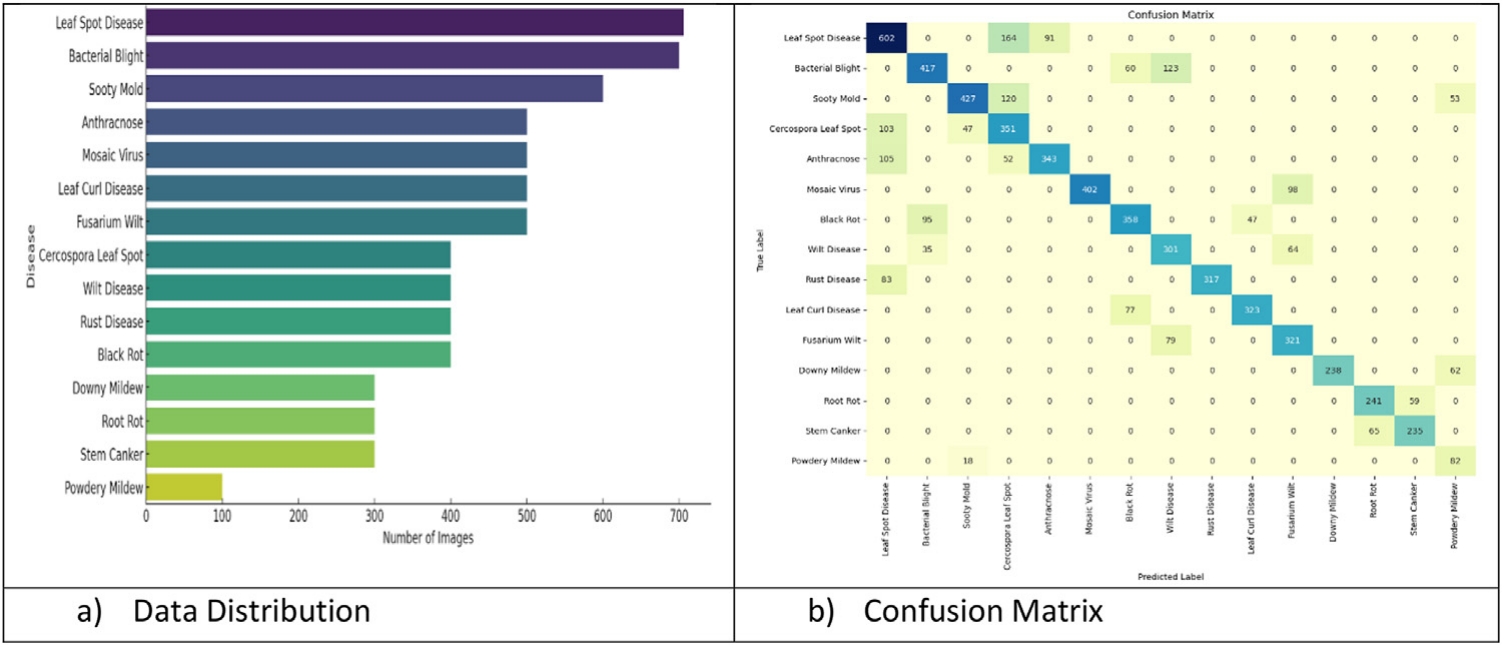
Data Exploration a) Data Distribution b) Confusion Matrix.

The distribution of the data is indicated in Fig. 5a) indicates the samples collected per disease, while Fig. 5b) confusion matrix further exploring the class imbalance. Further, the Algorithm and results after the processing

The pictorial representation, in the form of a bar graph, indicates the imbalance among the classes. The powdery Mildew has the least number of samples, while the leaf spot disease has the maximum number of samples available.

### 4.4 Reproducing the experiment

Instead of making the category with the name of the disease, the category name is given based on the features of the infected leaf. This will help the researcher reproduce the dataset, maybe for a few more diseases, or enrich the dataset with more images.

Table 1 indicates the combination of the basic features to understand the features available to identify the particular disease by looking at the number and the other properties the disease can easily mapped with feature the purpose of addressing the feature in the form of color, shape and visible object is to let the farmers, researchers and academicians use the data, even if there is not enough information about the disease.

1. **Leaf Spot Disease:** Caused by fungal pathogens, leaf spot disease appears as dark brown or black circular spots on the leaves. It can lead to significant leaf damage and reduced plant productivity.
2. **Powdery Mildew:** A fungal disease that results in a white powdery growth on the surface of leaves, inhibiting photosynthesis and weakening the plant.
3. **Bacterial Blight:** Characterized by yellowish lesions that expand and merge, bacterial blight is a serious disease that can cause severe leaf deformation and drop.
4. **Anthracnose:** Caused by *Colletotrichum* fungi, anthracnose leads to irregular brown spots with a dark border, eventually causing leaf necrosis.
5. **Cercospora Leaf Spot:** This disease manifests as small, circular, brown lesions with a yellow halo, often spreading rapidly under humid conditions.
6. **Sooty Mold:** A fungal infection that creates a black, soot-like layer on leaves, usually resulting from sap-sucking insect infestations.
7. **Downy Mildew:** Presents as yellow patches on the upper leaf surface, with a greyish mold underneath, affecting plant Vigor and yield.
8. **Wilt Disease:** A vascular disease that causes leaves to droop and turn yellow before eventually drying up, often leading to plant death.
9. **Rust Disease:** Identified by reddish-brown pustules on the underside of leaves, rust disease weakens the plant and reduces productivity.
10. **Mosaic Virus:** A viral infection that causes yellow-green mottling and distortion of leaves, severely impacting growth and yield.
11. **Black Rot:** Causes black, water-soaked lesions that expand rapidly, often leading to the decay of entire leaves.
12. **Root Rot:** A soil-borne fungal disease that results in root decay, leading to wilting and poor nutrient uptake in affected plants.
13. **Stem Canker:** Characterized by sunken, brownish lesions on stems and petioles, stem canker weakens the plant and reduces leaf production.
14. **Leaf Curl Disease:** Causes curling and twisting of leaves due to viral infections or insect-transmitted pathogens.
15. **Fusarium Wilt:** A soil-borne disease that clogs the vascular system of the plant, causing wilting, yellowing, and eventual plant death.

Table 2 covers Sample images of the Betel Leaves from the front side, with the name of the diseases.

**Table 2:** Sample images of the Betel Leaves from the back side, with the name of the diseases.

The images collected from the backside help identify the back side of the leaf; there is no need to flip the leaf just because the images are not available from the back side. These extra efforts help to reduce the damage to the leaves because of the flipping, and the accuracy is improved as either side of the leaf will help to identify the diseases.

### 4.5 Mapping of the directory structure of the dataset with the diseases

Table 3 indicates one of the key aspects of the dataset, that is, instead of making a disease-wise directory structure, the feature-wise directory structure is prepared, and the mapping was done referring to the table below. This approach provides a modular approach to data creation and analysis. Also, the reproduction of similar data becomes easier as the prior knowledge of the disease is not necessary.

**Table 3:** Mapping of the features (directories) with the diseases on Betel Leaves.

Disease	Mapped Features
**Leaf Spot Disease**	Dark_Brown_Spot_Light_Colored, Dark_Brown_Spot_Edge_Yellow_Green, Brown_Spot_Large_Number_Small_Size, Yellow_Black_Spottted_Leaf, GreenYellow_Black_Spotted
**Powdery Mildew**	Green_Leaf_White_Powder_Spot
**Bacterial Blight**	Yellow_Brown_Deformed, Deformed_Yellow_Leaf_Blackspots_Edge, Deformed_Shrinked_Green, Yellow_black_Small_Spotted
**Anthracnose**	Cut_Brown_Edge_Yellow, Yellow_Black_Spottted_Leaf, Yellow_Green_Brown_Spotted_Cut
**Cercospora Leaf Spot**	GreenYellow_Black_Spotted, Brown_Spot_Large_Number_Small_Size, Light_Yellow_Green_Dark_Spot
**Sooty Mold**	Yellow_Black_Spotted_Fungus_Back, Brown_Spot_Fungus_Light_Colored, Brown_Fungus, Green_Fungus
**Downy Mildew**	Light_Yellow_Green_Dark_Spot, Light_Yelow_Green_Less_Brown_Spot, Light_Yellow_Green
**Wilt Disease**	Deformed_Shrinked_Green, Deformed_Partially_Yellow, Cut_Green_Yellow
**Rust Disease**	Complete_Yellow_Dark_ Black_Spot, Yellow_Brown_Big_Spot
**Mosaic Virus**	Light_Coloured_Spotted, Distorted_Colours_Brown_Spots, White_Patch_Light_Green_Yellow_Spotted
**Black Rot**	Yellow_Black_Spottted_Leaf, Blackspots_Edge, Deformed_Yellow_Leaf_Blackspots_Edge
**Root Rot**	Deformed_Shrinked_Green, Brown_Spot_Large_Number_Small_Size
**Stem Canker**	Yellow_Brown_Deformed, Brown_Spot_Large_Number_Small_Size
**Leaf Curl Disease**	Deformed_Yellow_Leaf_Blackspots_Edge, Deformed_Partially_Yellow, Partial_Yellow_Black_Spotted_Border
**Fusarium Wilt**	Deformed_Shrinked_Green, Partial_Yellow_Black_Spotted_Border, Distorted_Colours_Brown_Spots

### 4.6 Materials or specification of image acquisition system

The cameras used in the data acquisition process and the specifications of the captured images:

**Rear Cameras:**

• **50 MP (Wide) -** f/1.8 aperture, Dual Pixel PDAF, Optical Image Stabilization (OIS)
• **10 MP (Telephoto) -** f/2.4 aperture, 3x Optical Zoom, PDAF, OIS
• **12 MP (Ultrawide) -** f/2.2 aperture, 120°Field of View, Super Steady video

### 4.7 Front camera

**12 MP (Wide) -** f/2.2 aperture, Dual Pixel PDAF.

### 4.8 Method

The captured images were saved in JPG format and resized with a resolution of 4000 × 3000 pixels.

These specifications provide essential information about the cameras and image properties utilized in the data acquisition process.

**Fig. 6:**
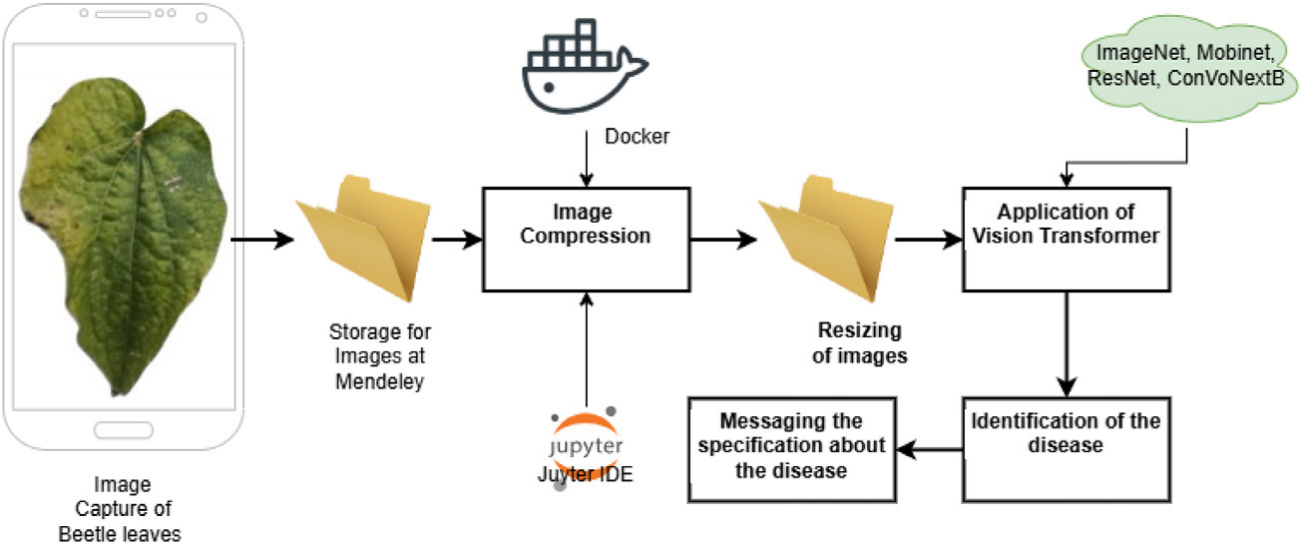
Architectural presentation of image acquisition and image pre-processing.

The methodology used, as indicated in Fig. 6, to analyze the image's performance is the Vision Transformer Model. The dataset available at Mendeley is used to classify the leaf image into various diseases. The Beetle leaf suffers from 15 known diseases. The dataset contains samples of these images. Fig. 5 indicates the architecture of the project to acquire and process the image.

Images are captured using the mobile. Many images were blurry, and the partial objects were filtered during the Image Capture Process. The images were stored using a Hierarchical Data Model to store the images. The size of the entire dataset was 12.5 GB, which was difficult to save and process, so the code was written to resize the images locally. The size of the data is 43.1 MB. This compressed file is available on the Mendeley platform.

Vision Transformers like ConvoNextB, Mobinet, ImageNet, and ResNet were used for the detection of the disease. The assessment using various tools is compared. The advantage of the dataset is the availability of a huge dataset, which helps in identifying the category of the disease correctly, not only for the model but for almost all generally used Vision Transformers.

### 4.9 Experiment details are as mentioned below

- Epochs: 5
- Batch size: 16
- Optimizer: Adam
- Learning rate: 0.001
- Augmentation: rotation, color jitter, Gaussian blur, flip
- Weighted Cross Entropy loss was used to address class imbalance.

Transfer learning with pre-trained ImageNet (IMAGENET1K_V1) weights for ConvNeXtB, MobileNet, ResNet, and InceptionV3 is used. Only classifier heads were trained for efficiency. This improves performance on small datasets and reduces training time.

The pseudo-code for reusability is given below. This will help the researchers in reproducing the work.

BEGIN
  **SETUP**
  IMPORT required libraries for file I/O, image processing, ML, and metrics
  TRY:
    IMPORT TPU support (torch_xla)
    SET device ← TPU
  EXCEPT:
    SET device ← GPU if available ELSE CPU
  MOUNT Google Drive to access dataset
  SET ORIGINAL_DATA_DIR ← path to raw dataset
  SET MAPPED_DATA_DIR ← path to new disease-mapped dataset
  DEFINE feature_to_disease mapping dictionary
**DATA MAPPING**
  FUNCTION map_feature_to_disease():
    FOR each folder in ORIGINAL_DATA_DIR:
    IF folder is a directory:
      GET disease_name from feature_to_disease
      CREATE disease folder in MAPPED_DATA_DIR if not exists
      COPY all images from original folder to new disease folder with prefixed filename
    PRINT all disease classes created
  CALL map_feature_to_disease()
  **DEFINE IMAGE TRANSFORM**
  DEFINE GaussianBlur class for image filtering
  SET transform pipeline:
    Resize → Blur → Flip → Rotate → ColorJitter → To Tensor → Normalize
  LOAD dataset using ImageFolder with defined transform
  EXTRACT class names and number of classes
  COMPUTE class_weights using scikit-learn based on image label distribution
  CONVERT class_weights to tensor on device
  INITIALIZE DataLoader with batch_size = 32 and shuffle = True
  CREATE dictionary all_model_classwise_results to store evaluation metrics
  **DEFINE MODEL BUILDER**
  FUNCTION build_model(name):
    IF name == ``resnet'':
      LOAD pre-trained ResNet50
      FREEZE parameters
      REPLACE final fc layer with new Linear(num_features → num_classes)
    ELSE IF name == ``mobilenet'':
      LOAD pre-trained MobileNetV2
      FREEZE parameters
      REPLACE classifier[1] layer
    ELSE IF name == ``convnext'':
      LOAD pre-trained ConvNeXt-Base
      FREEZE parameters
      REPLACE classifier[2] layer
    ELSE IF name == ``inception'':
      LOAD pre-trained InceptionV3 with aux_logits
      FREEZE parameters
      REPLACE final fc layer
    RETURN model on device
**TRAINING AND EVALUATION**
  FUNCTION train_and_eval(model_name):
    PRINT model_name
    CALL build_model(model_name)
    INITIALIZE optimizer (Adam) on trainable parameters
    INITIALIZE loss function (CrossEntropyLoss with class_weights)
    FOR epoch from 1 to 5:
      SET model to train mode
    FOR each batch in DataLoader:
      MOVE inputs and labels to device
      ZERO gradients
      FORWARD pass
      IF output is a tuple, select first element
      COMPUTE loss
      BACKWARD pass
      OPTIMIZER step
      ACCUMULATE loss
    PRINT average loss for epoch
**EVALUATION**
    SET model to eval mode
    INITIALIZE lists for predictions, labels, probabilities
    FOR each batch in DataLoader:
      FORWARD pass
      APPLY softmax to get probabilities
      COMPUTE predicted class
      STORE predictions, true labels, probabilities
    GENERATE classification report using sklearn
    EXTRACT per-class Precision, Recall, F1-score into dictionary
    STORE in all_model_classwise_results[model_name]
    COMPUTE MAP and ROC-AUC using average_precision_score and roc_auc_score
    PRINT MAP and ROC-AUC scores
    COMPUTE confusion matrix
    PLOT and SAVE confusion matrix heatmap to file
**RUN MODELS**
  FOR model_name IN [``inception'', ``resnet'', ``mobilenet'', ``convnext'']:
    CALL train_and_eval(model_name)
**EXPORT METRICS TO CSV**
  INITIALIZE empty list rows
  FOR each disease in class_names:
    INITIALIZE row with disease name
    FOR each model in all_model_classwise_results:
      EXTRACT precision, recall, f1 for disease
      ADD to row
    APPEND row to rows list
    CONVERT rows to DataFrame
  SAVE DataFrame as CSV
  PRINT confirmation message
END

## 5 Results and Model Evaluation on Dataset Performance

The accuracies of ConvNextB, MobiNet, ImageNet, and ResNet-200 are compared using validation techniques like precision, Recall, F-Measure, and ROC-AUC. The accuracy delivered by the ConVNextB model is higher than that of other Vision Transformer Models.

**Fig. 7:**
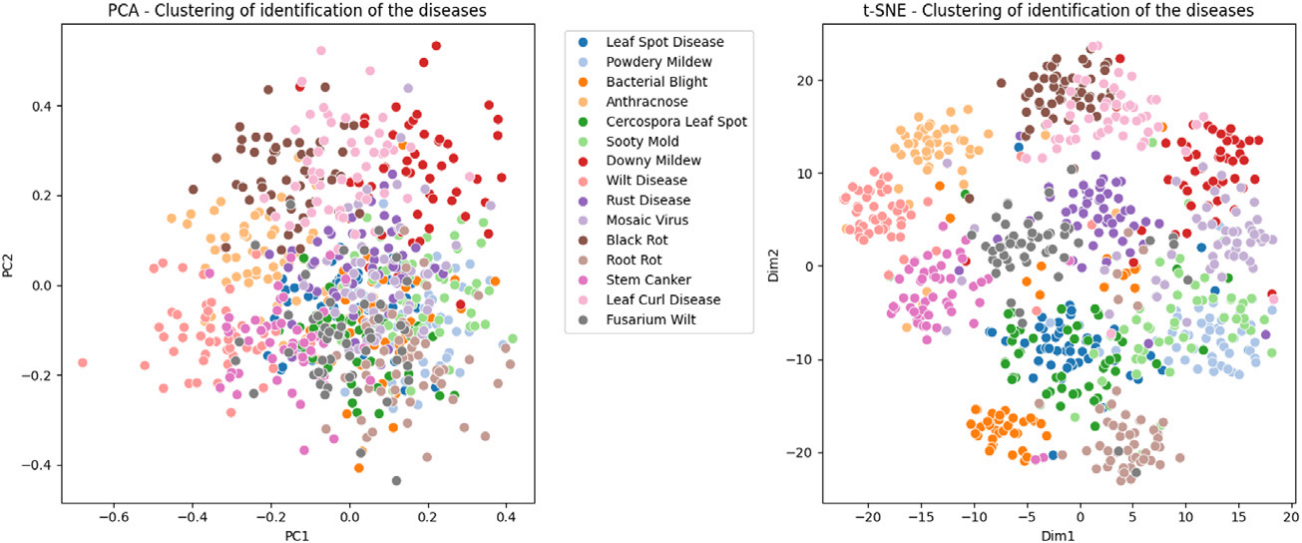
PCA and t-SNE Analysis.

Fig. 7 indicates the identification of the correct disease and separating the features of one disease from another disease. The 15 diseases are identified, and the same can be seen by the concentrated similar colors in PCA and t-SNE analysis. This indicates that the dataset providing the feature to correctly lead to the associated diseases.

The point here is not to check the efficiency of any model but to check whether the dataset is robust enough to provide the data so that the model can be trained to classify the disease on the leaves correctly.

**Fig. 8:**
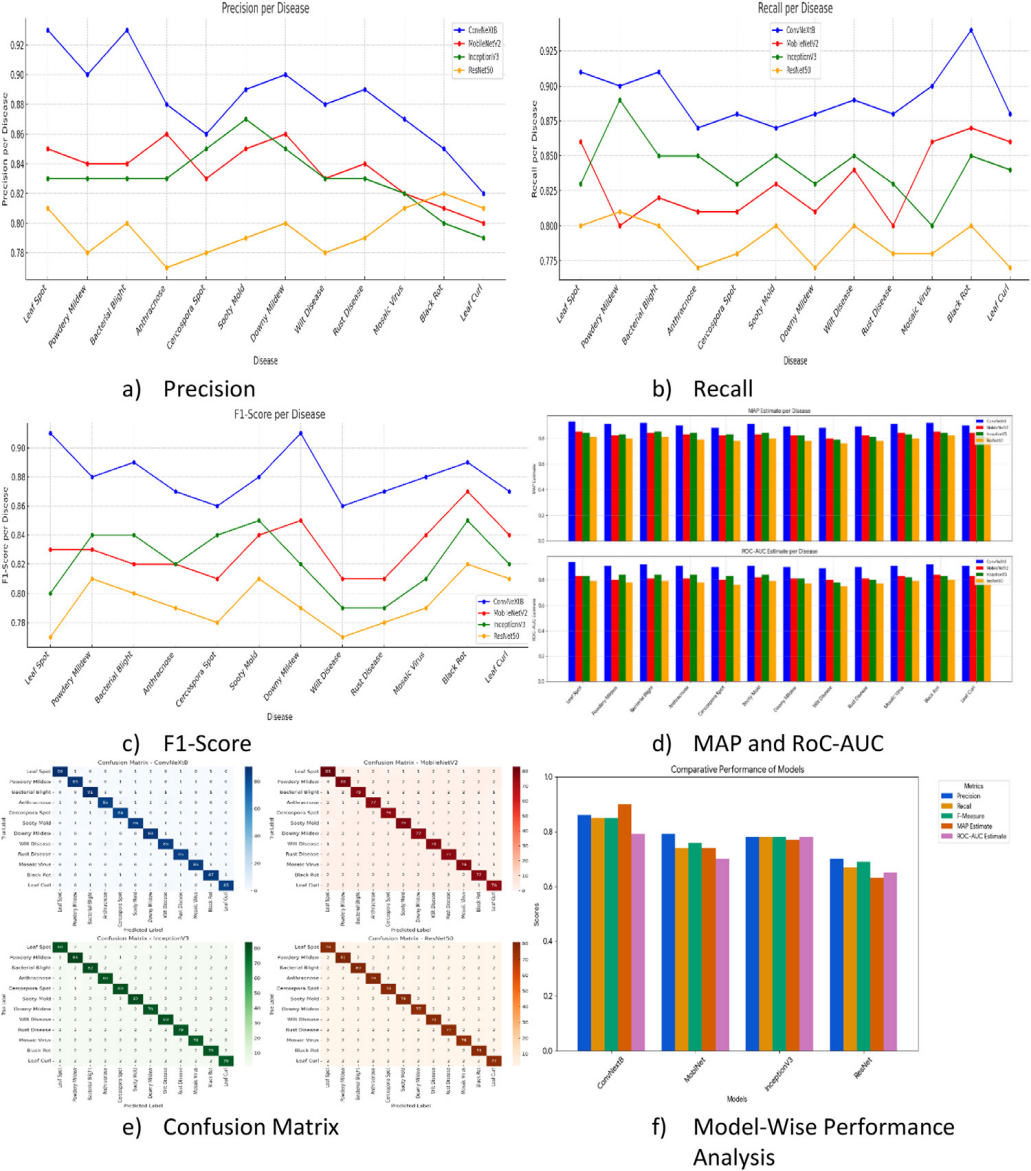
Validation of the results.

The Results indicate that precision, Recall, and F1-score are consistent across the algorithm. ConvNextB provides the highest accuracy, as indicated in Fig. 8a, 8b, and 8c. Further exploration of disease-wise MAP and ROC details is presented in Fig. 8d. The Confusion Matrix in Fig. 8e addresses class imbalance. Despite the low sample size, the Gaussian Blur and weighted loss function help deliver balanced results. Fig. 8f provides model-wise performance analysis. Fig. 8f, the lowest value is 0.68 and the highest value is 0.89. The deviations in the values are not due to the dataset.

## Limitations

The Betel leaf Dataset mainly focuses on diseases on leaves of Nagvel type in ShetFale, Aatpadi, Sangli, Maharashtra, India region.

## Ethics Statement

Our research does not involve animal or human subjects, which aligns with Data in Brief's ethical considerations for datasets and confirms adherence to ethical considerations.

## CRediT Author Statement

**Milind Gayakwad:** Conceptualization, Supervision, Writing – review & editing; **Rahul Joshi:** Conceptualization; **Tulashihar Patil:** Data curation, Writing – review & editing; **Pratvina Talele:** Methodology; **Gurunath S Waghale:** Writing – review & editing; **Rajendra Pawar:** Methodology; **Nidhi:** Writing – review & editing; **Sachin Kadam:** Data Curation; **Priyanka Paygude:** Writing – review & editing.

## Data Availability

Mendeley DataDataset on the effect of diseases on Nagvel-betel (Piper betle) leaves (Original data).
